# Does it actually feel right? A replication attempt of the rounded price effect

**DOI:** 10.1098/rsos.171127

**Published:** 2018-04-25

**Authors:** Christopher Harms, Hanna A. Genau, Carolin Meschede, André Beauducel

**Affiliations:** Department of Psychology, University of Bonn, Bonn, Germany

**Keywords:** product pricing, consumer psychology, rounded price effect, replication

## Abstract

How does the roundedness of prices affect product evaluations? The ‘rounded price effect’ postulates that depending on the context, rounded or non-rounded prices increase the purchase likelihood of consumers. The study presented here is a replication attempt of these findings and the proposed mediation of the effect through a sense of ‘feeling right’ when evaluating the product. *p*-Curve analysis and the R-Index are used to assess the robustness of the originally reported statistics since original data were not available. A pre-registered replication of study 5 from the original article was conducted in a sample of *N*=588 participants. For both the original product and one alternative product neither an interaction between price roundedness and context, nor a mediation through ‘a sense of feeling right’ was found. Our results suggest that the effect is either smaller than originally reported or contingent on other, not investigated factors. Further studies might investigate contingencies in larger samples.

## 1. Introduction

When selling products and services to customers, companies have to decide on a reasonable price for their goods. While this is primarily driven by economic concerns on the part of the company, psychological factors also need to play a role because the customer’s buying decision is a psychological evaluation of the product’s properties including its price. Research on consumer behaviour and the effect of pricing on decision-making is thus a popular field for behavioural economics and marketing research. Several theories concerning product pricing have been proposed. In 2015, Wadhwa & Zhang [1] investigated how the roundedness of a price affects product evaluations and what moderates and mediates this relationship. They termed their findings ‘rounded price effect’ and built upon previous research on the perception of rounded versus non-rounded numbers. In a series of five experiments they report the rounded price effect using different manipulations and dependent variables. In their fifth and final experiment, they report a mediation of the effect through a proposed sense of ‘feeling right’ when evaluating the product.

In recent years, studies from nearly all subfields of psychology have been under increased scrutiny in the context of the ‘replication crisis’ [2–5]: as several studies suggest, we cannot take reported effects in the scientific literature at face value. As the findings by Wadhwa and Zhang have practical relevance to marketers, independent replication of the effect and a reasonable estimation of its size are desirable. From the theoretical outline of the effect one can expect the effect to be contingent on various factors. As a first step towards a better understanding of these external influences on the effect, a close replication under the same—or at least very similar—conditions as in the original study is warranted.

The article is structured as follows: an overview on the five experiments in Wadhwa & Zhang [1] is given in the next section. We consider the fifth study reported in Wadhwa & Zhang [1] as particularly relevant as it investigates both the rounded price effect and the proposed mediation through a sense of ‘feeling right’ as a theoretical explanation of the effect. ‘Feeling right’ hereby refers to an experience of engagement in the subject that is triggered through the fit of price roundedness and decision context—a theorized mechanism based on regulatory-fit theory [6,7]. This leads us to the planning of the present study: a replication attempt of the fifth study from Wadhwa & Zhang [1] with a focus on both the rounded price effect and the mediation through ‘feeling right’.

After reiterating the main findings of the original study in the next section, our article covers two approaches: first, we will use statistical techniques to investigate the findings for replicability and, second, we will report the results of an attempted direct replication of study 5 from the original paper.

## 2. The rounded price effect and ‘feeling right’

Wadhwa & Zhang [1] base their rounded price effect on previous research on the perception of rounded versus non-rounded numbers [8–12]. Since rounded numbers are more prevalent in everyday communication and are more fluently processed, it is argued that rounded numbers increase the reliance on an emotional evaluation of the subject, i.e. rounded prices (e.g. 40.00 euros) lead customers to stronger rely on their feelings when evaluating a product. The opposite proposition, that non-rounded numbers (e.g. 31.22 euros) increase a reliance on thoughts and cognitive judgement, is also stated. Applied to the context of consumer decision-making, the authors hypothesize that the context of a decision (cognitive versus emotional) will determine the reliance on either feelings or cognition and lead to an intensified judgement depending on the roundedness of the price. They state, that, if consumers are in an emotional context (i.e. buying a product for personal pleasure or, e.g. a vacation), products are more positively evaluated if the price is rounded (e.g. 80.00 euros), as rounded numbers are more fluently processed in this case. By contrast, if consumers are in a cognitively driven context (i.e. buying a product for school or work), products will be evaluated better if the price is non-rounded (e.g. 81.43 euros).

This effect, termed ‘rounded price effect’, is hypothesized to be mediated through ‘a sense of feeling right’ [1], p. 1174. This is concluded from findings, that the fit between two factors generally ought to engage subjects based on regulatory-fit theory [7,13]. Fit in the current study is given when the context is emotional and the price is rounded and vice versa.

Studies 1–3 in Wadhwa & Zhang [1] are designed to test the predictions of the rounded price effect. Decision contexts in studies 1 and 2 are varied through the instructions of the study (participants were asked to imagine buying a product for, for example, either a family vacation or a school project) and prices are presented either as rounded or non-rounded numbers. Study 3 manipulates available processing resources through a cognitive memory task. While studies 1 and 3 test the interaction between roundedness and context or processing resources, respectively, on the outcome variable ‘purchase intention’, study 2 tests the effect on the ‘anticipated purchase satisfaction’ and an evaluation of the ‘perceived quality of pictures’ purportedly taken with the product (a digital camera). Study 4 tests the rounded price effect when priming participants to either rounded or non-rounded numbers. In study 5, finally, participants are primed using questions demanding either cognitive resources (through a simple calculus task) or questions on emotional reactions towards different words. The last study also investigates the proposed mediation through the sense of feeling right evoked in the participants.

All the five studies provide evidence for both the proposed rounded price effect and the ‘feeling right’ mediation. The effect is described to be generally dependent only on context and price roundedness and the authors do not state any argument for a limited universality of the effect. Further research thus may answer several different questions. First is the effect replicable when using the exact same stimulus material and the exact same population from which a sample is drawn? Second, does the effect generalize across different populations and different varieties of stimulus material?

The original article does not present limitations to the effect, thus some universality of the findings is implied. Since the original material, however, was not publicly available when the study was conducted, a direct replication using the exact same material was not possible.^1^ Further, no details on the nature of the original sample are reported except for the online tool used to sample participants (Amazon MTurk). In this case a direct replication, i.e. a replication using the same stimuli and a very similar sample, is unfeasible [14]. A close replication using materials based on the reported method, however, is possible and was done for the present study as we outline below.

Before we report our replication attempt of study 5, we will analyse the original findings for possible effects of different biases [15,16] for all experiments reported by Wadhwa & Zhang [1].

## 3. Analysis of the original findings

Several replication projects and meta-analytical investigations have shown that effect sizes in the published literature, generally, cannot be taken at face value. Especially, the adverse effects of researchers degrees of freedom [17] or the garden of forking paths [18] as well as publication bias [19] have recently been discussed in the context of replicability of research findings. Especially, studies which have not been pre-registered are susceptible to report inflated effect sizes due to publication bias, selective reporting and other common research practices [17,20]. Since the original study by Wadhwa & Zhang [1] was not pre-registered and did not provide material, data or analysis scripts openly, we first checked the reported results using meta-analytical procedures. Thereby, we did not presume that the original study is biased but checking indicators of potential bias should be performed as a routine for any study, especially when a replication study is intended. The approaches outlined below can provide value information for the planning of a study, for example in the context of a power analysis.

In order to estimate the replicability of the effect, we used *p*-curve analysis [16,21] and Schimmack’s [22] R-Index.

### 3.1. *p*-Curve analysis

Simonsohn *et al.* [21] observed that there is an incentive for researchers to report significant effects and that through rather simple and partly commonly used techniques *p*-values can be moved under the threshold of 0.05; they termed these techniques cumulatively *p*-hacking.^2^ Based on the statistical fact that, under the null hypothesis of no effect, observed *p*-values are uniformly distributed between 0 and 1, it can be concluded that the distribution of *p*-values is skewed when the null hypothesis is practically true and such techniques are used (e.g. selectively reporting variables and covariates, arbitrary removal of outliers; see [23] for an overview). With the *p*-curve, Simonsohn *et al.* [21] introduced a meta-analytic approach aimed to identify studies that have either been selectively reported based on the significance of the statistical tests or been ‘*p*-hacked’ [17] to reach statistically significant results. It is based on the observation that, in a given set of studies, finding many *p*-values close to a level of significance of 0.05 is rather unlikely in true and unbiased datasets. In order to perform a *p*-curve analysis, the *p*-values significant at a level of 0.05 of a set of studies (in our case, the five studies in the original paper) related to the substantial theory of interest are entered into the analysis. The *p*-curve then plots the frequency of these *p*-values in bins and performs statistical tests on the shape of the curve. The resulting curve should be right skewed if the data contain *evidential value*, i.e. low *p*-values (e.g. *p*=0.001) would be reported more often than values close to 0.05 (e.g. *p*=0.048). In their revision of the approach [16], the *p*-curve is split in half and analyses are performed for the whole curve (*p*<0.05) and the half curve (*p*<0.025) to allow for a more sensitive analysis.

**Figure 1.**
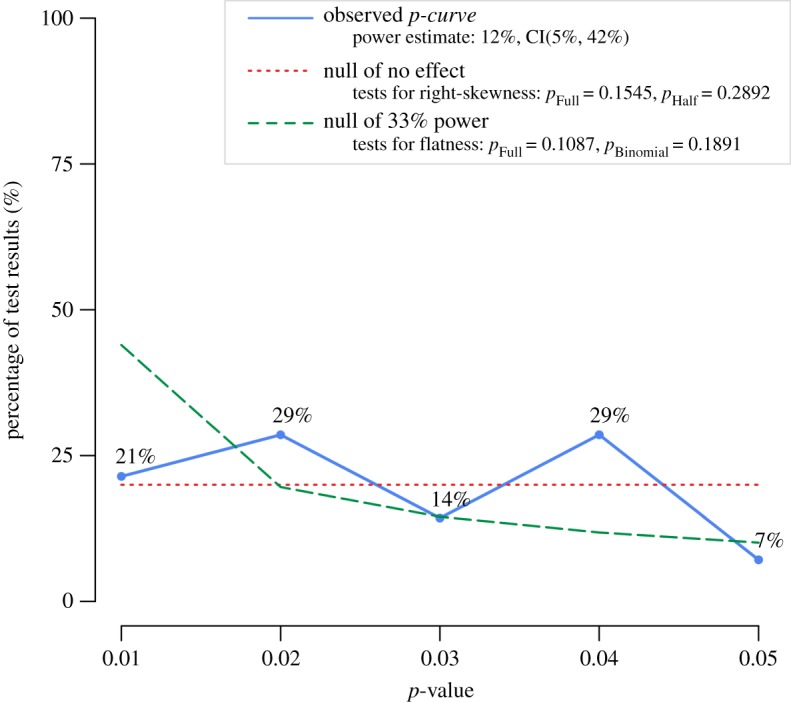
*p*-Curve analysis which does not indicate evidential value in the data of Wadhwa & Zhang [1]. See electronic supplementary material, appendix for a table detailing the selection of *p*-values. Note: the observed *p*-curve includes 14 statistically significant (*p*<0.05) results, of which 8 are *p*<0.025. There were 3 additional results entered but excluded from *p*-curve because they were *p*>0.05.

For the set of focal analyses in the paper by Wadhwa and Zhang, *p*-curve analysis does not suggest evidential value in the data (*z*=−1.02, *p*=0.155 for the full *p*-curve; *z*=−0.56, *p*=0.283 for the half curve); see figure 1 for details. For the 14 statistical tests selected in the analysis, the estimated statistical power is 11% (90% CI: [5%,42%]). Since the test for the flatness of the *p*-curve is also not significant (*z*=−1.23, *p*=0.109 for the full *p*-curve; *z*=2.51, *p*=0.994 for the half *p*-curve), the analysis is overall inconclusive. We cannot conclude that the data provide evidential value but also cannot conclude selective reporting.

While the analysis of a set of studies based on the likelihood of observed *p*-values can be a useful tool to evaluate the trustworthiness of findings in the published literature, the technical details of the approach as detailed by Simonsohn *et al.* [21] have also been criticized. Morey [24], for example, criticizes the choice of test statistics and the problem of grouping tests for the analysis.

### 3.2. Replicability index

Schimmack [22] has proposed the *replicability index* (R-Index) as a measure for replicability sensitive to selective reporting and the exploitation of ‘researchers degrees of freedom’. It is intended as an investigative analysis for statistical results and based on the (low) probability of a paper with multiple studies reporting only significant results [15]. If studies with given observed power (which is based on the realized sample size, the observed effect size and the resulting *p*-value) report a number of significant results, the probability of such a pattern can be calculated and is represented in the R-Index.

Using the Excel spreadsheet provided on the R-Index website, we calculated the R-Index for the same focal hypotheses tests as for the *p*-curve.^3^ With a proportion of significant results of 82.35% and a median observed power of 0.588, the R-Index for the paper by Wadhwa and Zhang is 0.352. Generally, the R-Index is closer to 1.0 for likelier patterns given the median observed power and smaller for unlikely numbers of significant results. The R-Index for the present case thus indicates that the proportion of 82.35% significant results (success rate) is rather unlikely given the median observed power of 0.588. Higher power or fewer significant results would have led to a higher R-Index.

Since the formulation of the R-Index procedure has not yet been published in a peer-reviewed journal there has not been a lot of discussion about the possibilities and limitations of the method. It is important to note that there is disagreement about how valid ‘observed power’ is for statistical inferences (e.g. [25]) and that the R-Index does not directly provide a statistical hypothesis test for replicability.

Neither of the two meta-analytic methods, *p*-curve and R-Index, can make a definitive statement about biases in published studies but they provide relevant information on how to plan a replication study. The results do not allow us to conclude that the studies have been reported selectively or results have been heavily skewed (consciously or unconsciously), but we also cannot conclude that the studies contain ‘evidential value’. In the light of the inconclusive results of the indicators of bias, a replication study may be especially important. In practice, the results and the well-known presence of publication bias in the literature lead us to be cautious about the size of the effect as reported in the original study: if the effect is present, it is probably smaller in size than reported in the original paper (see power analysis for the replication study below). These considerations also highlight the need for an independent, pre-registered replication to generally reduce biases in the literature about the effect under investigation.^4^

### 3.3. Sample of Wadhwa & Zhang [1]

The original sample was recruited through Amazon MTurk. Research on the quality of MTurk samples comes mostly to the conclusion that they are more socio-demographically diverse than common samples of psychology students and show satisfactory psychometric reliability [26–28]. On the other hand, recent studies about MTurk samples showed that they cannot be trusted in all cases as participants misreport their demographics [29], measured effects differ when compared to laboratory and other survey settings [30] and as data quality can be an issue when participants are not attentive to the presented tasks [31]. Further, MTurkers often participate in a high number of studies and it is unclear how this affects the results of studies conducted with MTurk samples [32]. Since Wadhwa & Zhang [1] did not report details on sample characteristics and demographics in their sample, it is difficult to evaluate how economically and culturally diverse the sample was when compared to other samples such as the one in our replication study below.

## 4. Replication study

Based on the findings of the original study by Wadhwa & Zhang [1], we planned a close replication study in order to independently investigate the proposed effect. To be in line with current recommendations on replication studies (e.g. [14]), we contacted the original authors asking for either the original dataset and materials or their comments on our study plan. Unfortunately, despite three emails to both authors, they did not respond to our inquiries prior to the study. Thus, the study design had to be based solely on the original article.^5^

We have pre-registered our replication attempt. Further, we provide all stimulus material, data and analysis scripts in an openly available online repository at https://osf.io/r942e/.^6^

As outlined above, study 5 used a priming task to prime participants for either an emotional focus or a focus on cognition. A digital binocular camera was presented with either a rounded or a non-rounded price.

A previous replication of study 3 from Wadhwa & Zhang [1] was performed by O’Donnell & Nelson [33]. This replication study was not published in a peer-reviewed journal but is available online at https://osf.io/am32k/ including study materials and data. We will include their results in the comparison of our estimates to the original study in the results section of our paper.

### 4.1. Pre-study

As our replication would be conducted in Germany and digital binocular cameras are a rather uncommon product here, we first conducted a pre-study evaluating the original product and the potential willingness to buy this product. We also compared it to a product that we expected to be more popular in our pre-study sample, namely an instant camera.

For this pre-study, 50 undergraduate psychology students at the University of Bonn had to rate the two products and state how much money they would be willing to pay for the products. To preclude a non-replication due to misunderstandings with respect to the product or its description, we included questions on whether the participants understood both the product and the advertising that were to be used in the replication study.

The results of the pre-study are reported in table 1. Ratings for comprehensibility were satisfactory. For both products, the willingness to pay was lower than the intended price for the manipulation (€80.00 and €81.43 versus an average reported price of e48.38 for the binoculars and e64.18 for the instant camera). The binoculars were not rated well by the pre-study participants, in general, but the instant camera was rated more favourably.

**Table 1.** Descriptive pre-study results.

	binoculars			instant camera		
variable	*M* (s.d.)	min	max	*M* (s.d.)	min	max
comprehension (advertising)	5.20 (1.969)	1	8	6.50 (2.063)	2	9
comprehension (product)	4.98 (2.005)	2	9	6.41 (2.207)	2	9
likelihood of purchase	2.14 (1.229)	1	6	4.86 (2.347)	1	9
willingness to pay	48.38 (51.337)	0.00	200.00	64.18 (49.095)	0.00	200.00

Since we were aiming to conduct a close replication study, we decided not to deviate too much from the original setting and included both products, despite the poor ratings of the binocular camera. As we feared that variance would be too small for any meaningful analysis we also included the instant camera as a second product in the replication attempt. We present the binoculars first and the instant camera second for all participants—while this might introduce carry-over effects, we were still able to investigate the original effect for the original product without an influence of the second product.

### 4.2. Method

Our pre-registered method is close to the approach used in the original study. Any differences from the originally reported study or our pre-registration are clearly explained in the following section.

#### 4.2.1. Power-calculation and sample

Based on the reported test statistics of the focal prime × roundedness interaction (*F*_1,314_=13.18) in the original article, an effect size estimate of *η*^2^=0.040 was calculated. While only 250 participants were required to achieve 90% power in a 2×2-design with an *α*-level of 0.05, based on the analyses of the original findings, we assume that the true effect size is very likely to be smaller than reported (see considerations above). We thus aimed for a sample of 600 participants, which is nearly twice the original sample size of 318. This would result in a power of 90% to detect an effect *η*^2^=0.017.

Using several different online channels, such as social networks and newsletters of online magazines, a convenience sample of 609 German-speaking participants was collected between 29 June 2016 and 19 August 2016. After finishing the experiment, participants could provide their email address to take part in a lottery to win one of three Amazon gift cards with a value of 20 euros each. Email addresses were stored independently from the experimental data to ensure anonymity. In the original study, participants from the USA were recruited through Amazon MTurk and paid an undisclosed amount for their participation.

As stated in the pre-registration, we checked responses for plausibility and excluded eight participants who did not diligently answer the priming task.^7^ We further excluded three participants who were under-aged and 10 participants who self-reported as not carefully responding to the questions. This results in a sample size of *N*=588 for statistical analysis. Twenty-nine participants were flagged because they took longer than 13.30 min to complete the experiment (based on the exclusion rules proposed by [34]). Results and interpretation do not differ for the samples including and excluding slow participants and the following sections thus report results only for the final *N*=588 sample including the slowest participants.

Participants are mostly female (408 participants, 69%), between 18 and 86 years old (*M*=32.066, *s*.*d*.=13.007, *median*=26.500) and educational attainment was above average (49.7% have a university degree). Possibly relevant to the research question, 239 participants (40.6%) reported to be currently employed and thus having a regular income.

#### 4.2.2. Material

Since we were not able to receive the original study materials prior to the study, we designed our own material based on the descriptions in the text and the referenced literature.^8^ For the priming, participants were asked either for their spontaneous feelings towards the words ‘baby’, ‘Angela Merkel’, ‘football world champion’, ‘refugee’ and ‘family’ or for the answer to simple calculus tasks. In the response from the original authors we received after the study was conducted, we found out that the original study did not only use words for the affective priming condition but also pictures in contrast to what is reported in the original article. We discuss this difference later in the discussion section of this article.

As planned in the pre-registration we added a second product, an instant camera, to the product used in the previous study, digital camera binoculars, based on the results of our pre-study.

#### 4.2.3. Procedure

Participants were invited to the study via a link to the online questionnaire. On the first page, participants were provided with general information on the study. As a cover, they were told that the study consisted in fact of two parts: in the first part (the priming task), participants should answer some questions for a new questionnaire (‘feeling’ group) or a new ability test (‘cognition’ group), that is developed at the authors’ department. The second part was introduced as a study on product evaluation.

Participants were randomly shown either the questions on feelings or the calculus tasks before randomly shown the products either with a rounded (€80.00) or a non-rounded price (€81.43). The advertisement for the digital camera binoculars was shown first, followed by the questions for the product evaluation. We included questions on the purchase likelihood, which was answered on a 9-point scale (‘very unlikely’ to ‘very likely’), and the anticipated satisfaction with the product after purchase, which was also answered on a 9-point scale (‘very unhappy’ to ‘very happy’). For the operationalization of ‘feeling right’ one question (‘How did you feel when rating the product?’) with two dimensions for the answer (‘It felt right’ and ‘It felt wrong’, both on a 9-point scale from ‘I disagree completely’ to ‘I agree completely’) were asked and combined to a single measure by taking the difference between the two answers. Questions on the ease of processing of the product information were also asked similar to the study in the original paper but are not included in the results as it was not the effect of interest of the replication.

Up to this point, the replication study is very close to the original study using similar material. Next, the advertisement for the instant camera was shown with the same rounded (€80.00) or non-rounded (€81.43) price, again followed by the questions on the product. In order to closely replicate the findings from the original study and have possible carry-over effects only affect the second product, the binoculars were always presented first. The study concluded with questions on demographics such as gender and age as well as further questions on mood and photography as a hobby. Those questions were included for possible exploratory analyses but are not reported extensively herein.

For the lottery of Amazon.de vouchers, participants could provide their email addresses after finishing the actual study. Addresses were stored separately to ensure anonymity.

#### 4.2.4. Statistical analyses

The analyses were performed in a similar manner as in the original paper, i.e. for the rounded price effect we evaluated the interaction between the Priming and the Roundedness condition on purchase likelihood using a two-way ANOVA and planned contrasts. The mediation of the effect through a sense of ‘feeling right’ was evaluated using mediation analysis [20,35].

In addition to the pre-registered analyses, we will report Bayesian hypothesis tests in the result section. Bayes factors have some advantages over *p*-values and have been increasingly advocated by some in psychological research, especially when a symmetrical measure of evidence is desired that is able to quantify evidence in favour of a null hypothesis (e.g. [36–38]).

Bayes factors aim to evaluate the relative statistical evidence of two hypotheses from a Bayesian perspective in a quantitative way [37]. The hypotheses are represented as two different statistical models. Given the prior odds of two competing models, that is the odds of the models before any data are seen, the Bayes factor gives quantitative information about how the odds should be shifted based on the provided data. If the probability of the models is interpreted as subjective belief in either model, the Bayes factor tells us how we should rationally change our beliefs from prior to posterior (Bayesian belief updating):

Posterior Odds=Prior Odds×Bayes Factor.

Bayes factors, usually denoted as *BF*_10_, are a quantitative measure of relative statistical evidence that can be directly interpreted from its numerical value. However, there are recommended boundaries to interpret the value of a Bayes factor (e.g. in [39], table 1): a Bayes factor larger than 3 is considered as ‘moderate evidence’, larger than 10 as ‘strong evidence’, larger than 30 as ‘very strong evidence’ in favour of the numerator model. A Bayes factor close to 1 indicates only ‘anecdotal’ evidence and can be interpreted as inconclusive, as the data are close to equally likely under both hypotheses. The Bayes factor in favour of the denominator model is simply *BF*_01_=1/*BF*_10_, so the bounds can also be similarly applied in the other direction.

In order to calculate a Bayes factor, one needs to specify a prior distribution for the parameters involved. There are several ways to specify a prior distribution. If information on the parameters were available (e.g. domain knowledge), those could be used to derive a prior distribution. However, ‘default priors’ have been introduced for several common test scenarios which have been proposed based on their mathematical properties and their usefulness in the context of psychological experiments [37].

For the tests performed in the original study, Bayes factor alternatives were used additionally, i.e. when a traditional *t*-test was used in the original study, we also used the corresponding default Bayes factor for *t*-tests [40] and likewise for ANOVAs [41]. For the mediation analysis, in particular, we used the Bayes factor for mediation proposed by Nuijten *et al.* [39]. As some Bayes factors have to be estimated through simulation methods, Bayes factors which cannot be numerically calculated are reported with a proportional error estimate.

Further, to evaluate the success of the replication attempt, we used different approaches, including those mentioned in the pre-registration (vote-counting based on *p*-values and effect sizes, confidence intervals and the ‘small telescopes’ approach [42]) and replication Bayes factors [43,44]. Each approach is explained briefly below.

### 4.3. Results

First, we report the results for the rounded price effect and the mediation analysis. We will then summarize the success of the replication attempt from several perspectives. Lastly, exploratory results are reported that go beyond what was covered in the original study.

#### 4.3.1. Rounded price effect

**Figure 2.**
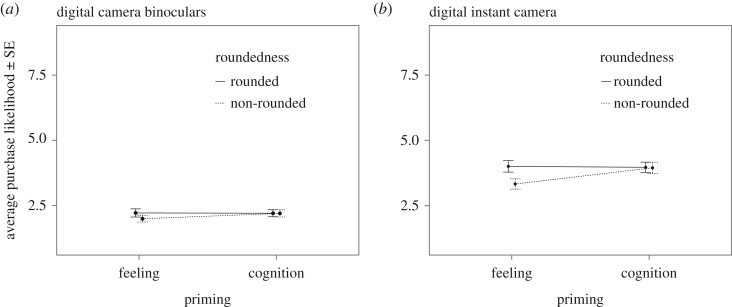
Interaction plots for the replication study. (*a*) The interaction plot for purchase likelihood of the digital camera binoculars—the same product as in the original study—while (*b*) shows the same interaction plot for the instant camera. Neither main effects nor interactions were significant. Planned contrasts reveal a significant difference between rounded and non-rounded prices in the feeling condition for the instant camera only. Purchase likelihood was rated on a 9-item scale. Error bars show standard error.

The rounded price effect should be visible as an interaction between the two between-subjects conditions, Prime and Roundedness. In our sample, the interaction was not significant for either the digital binoculars (*F*_1,584_=0.608, *p*=0.436; *BF*_10_=0.003±1.6% for the full model compared with an intercept-only model) nor for the instant camera (*F*_1,584_=2.526, *p*=0.113; *BF*_10_=0.039±2.2% again for the full model compared with an intercept-only model). See figure 2 for the binoculars and the instant camera, respectively. Planned contrasts did not reveal significant differences for either the feeling (*t*_584_=1.132, *p*=0.258; *BF*_10_=0.172) or the cognition condition (*t*_584_=0.028, *p*=0.978; *BF*_10_=0.092) for the digital camera binoculars. For the digital instant camera, on the other hand, the difference between rounded and non-rounded numbers was significant for participants primed with feelings (*t*_584_=2.322, *p*=0.021). While the difference is in the predicted direction, this presents only ‘anecdotal’ evidence from a Bayes factor perspective (*BF*_10_=1.653≤3). For participants solving the calculus tasks (cognition condition), the difference was not significant (*t*_584_=0.071, *p*=0.943; *BF*_10_=0.129).

The main effects did not reach significance; neither for the original product (Priming: *F*_1,584_=0.488, *p*=0.485, *BF*_10_=0.118; Roundedness: *F*_1,584_=0.670, *p*=0.413, *BF*_10_=0.129) nor for our alternative product (Priming: *F*_1,584_=2.028, *p*=0.155, *BF*_10_=0.259; Roundedness: *F*_1,584_=2.856,*p*=0.092, *BF*_10_=0.382). Since the evidence for a single main effect of Price Roundedness on the purchase likelihood for the instant camera was inconclusive considering the Bayes factor, more data might be necessary to rule out this effect entirely. However, the evidence was strongly in favour of the intercept-only model against a model containing both main effects for the instant camera (*BF*_10_=0.094±1.14%).

#### 4.3.2. Mediation analysis

The mediation analysis investigates if the fit between roundedness of the price and the cognitive or emotional context induces a ‘subjective experience of “feeling right” [that] will mediate the rounded price effect’ [1], p. 1181. To this end, mediation analysis [20] was used by the original authors and tested using both Sobel’s test and Preacher & Hayes’ [35] recommended bootstrapping approach. The latter is useful when raw data are available and yields more robust estimates as it does not assume a symmetrical sampling distribution for the mediated effect.

In line with the procedure in Wadhwa & Zhang [1], the independent variable of the mediation was a binary variable representing the fit between Prime and Roundedness conditions. That is, fit equals 1 for participants in the ‘Feeling + Rounded’ and ‘Cognition + Non-rounded’ conditions and 0 for everyone else. Considering this set-up, the analysis is a moderated mediation analysis where the moderation is coded in the independent variable.

**Figure 3.**
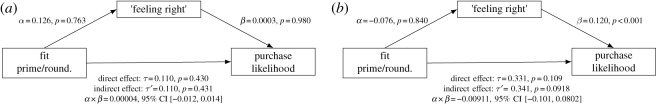
Analysis of moderated mediation [20] for the purchase likelihood of both products. (*a*) Digital camera binoculars and (*b*) instant camera. Reported are unstandardized regression coefficients and an effect size estimate for the indirect effect *α*×*β* with a bootstrapped 95% CI. Moderation is present in the analysis through the binary coded independent variable ‘fit’, which is 1 for priming on feeling and rounded prices and for priming on cognition and non-rounded prices.

Figure 3 shows the regression coefficients and reports the estimated indirect effect *α*×*β* for the replication study. Sobel’s tests were non-significant (binoculars: *z*=0.025, *p*=0.980; instant camera: *z*=−0.202, *p*=0.840).

Default Bayes factors for mediation using JZS priors [39] support the conclusion that there is statistical evidence that no mediation through ‘feeling right’ exist for either product (binoculars: BF_*m*0_=0.001; instant camera: BF_*m*0_=0.034), since the Bayes factors are strongly in favour of the null model when compared to the mediation model.

#### 4.3.3. Evaluation of replication success

**Figure 4.**
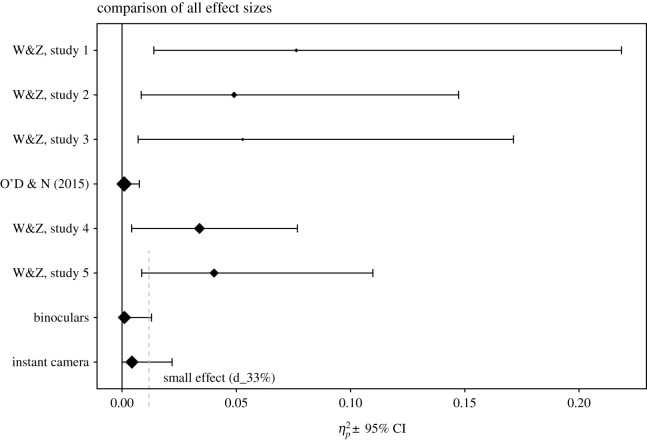
Forest plot for comparison of effect sizes across studies. Replications had higher power and show consistently smaller effect size estimates. Error bars are 95% CIs for effect sizes. Black line is effect size of zero. Dashed line indicates a ‘small effect’ [42] of $\eta_{\left(p;33\%\right)}^{2}=0.012$ for study 5 and our replications. Size of points are proportional to sample sizes in each study.

Based on the *p*-values for the interaction and planned contrasts we were not able to replicate the rounded price effect found by Wadhwa & Zhang [1] unambiguously, despite the descriptive statistics going in the same direction. Considering the estimated effect sizes and their respective 95% CIs (figure 4), the replication attempts have consistently smaller effect size estimates and higher power (including [33], replication attempt of study 3). All replication confidence intervals for $\eta_{p}^{2}$ include zero. In sum, this reduces our confidence in the presence of the proposed rounded price effect while an existing albeit very small effect cannot be ruled out.

Simonsohn [42] suggested comparing a replication attempt to an effect size that the original study had a power of 33% to detect (‘small telescopes’ approach). In the case of study 5 from Wadhwa & Zhang [1], this ‘small effect’ is $\eta_{\left(p;33\%\right)}^{2}=0.012$. In other words, the original study had 33% power to detect an effect of $\eta_{p}^{2}=0.012$.

As is visible in figure 4, both our two replication attempts and the original studies’ confidence interval include the ‘small effect’. Thus again, all studies are consistent with an existing, albeit very small effect.

The replication Bayes factor compares the hypothesis of an existing effect that is replicated in replication study with the hypothesis of a non-existent effect [43–45]. For the replication hypothesis, the data from the original study are used as prior information for the replication study. The replication Bayes factor is thus close to the above-mentioned concept of Bayesian belief updating.

The replication Bayes factor for the interaction term is BF_*r*0_=0.009 for the digital camera binoculars and BF_*r*0_=0.087 for the instant camera, indicating strong evidence in favour of the null hypothesis for both products: That is, the model stating an effect size of *f*^2^=0 for both studies is about 100 times and about 11 times, respectively, more likely than the model with the prior from the original experiment, under the assumption of balanced groups in both the original and the replication study [43]. Considering the significant difference between rounded and non-rounded prices in the feeling condition for the instant camera, the replication Bayes factor is BF_*r*0_=4.775 and shows evidence in favour of a successful replication for this singular contrast.

#### 4.3.4. Exploratory results

In the section above, we have reported our results separately for each of the two tested products. In a mixed design ANOVA, we tested any interaction between the product and the two between-subjects conditions Priming and Roundedness. Since participants in the pre-study already reported higher purchase likelihoods for the instant camera, it is consistent that in the analysis of the main study the effect of the product is significant (*F*_1,584_=235.528, *p*<0.0001). All other effects are non-significant, including the interaction of Priming and Roundedness across both products (*F*_1,584_=2.464, *p*=0.117) and the three-way interaction between Priming, Roundedness and Product (*F*_1,584_=1.034, *p*=0.310).

When including other collected variables in the mixed design ANOVA such as demographic variables, it reveals significant main effects for Age (*F*_1,572_=25.294, *p*<0.0001) and how often participants take photos in their free time (*F*_1,572_=8.807, *p*=0.003) on purchase likelihood (across both products), as well as a significant three-way interaction Priming × Roundedness × Photography in leisure time (*F*_1,572_=6.937, *p*=0.009). These results, however, cannot be understood in any confirmatory way as long as no theoretical underpinning gives rise to a sensible interpretation and the findings are replicated in a pre-registered study.

In study 2 of the original paper, ‘Anticipated Satisfaction’ was used as a dependent variable instead of Purchase Likelihood. For the replication, we have included Anticipated Satisfaction in the questionnaire and again used mixed design ANOVA to exploratively investigate the rounded price effect: again, both the two-way interaction Priming × Roundedness across both products (*F*_1,584_=3.307, *p*=0.070) and the three-way interaction Priming × Roundedness × Product (*F*_1,584_=0.282, *p*=0.596) failed to reach significance. Even if the two-way interaction is considered ‘marginally significant’, this result of an exploratory *post hoc* analysis constitutes again only extremely weak support for a very weak effect.

## 5. Discussion

Our results did not unambiguously support the rounded price effect and provide evidence against the proposed mediation. While the descriptive statistics hint in the predicted direction, analyses using significance tests and Bayes factors could not yield strong evidence for the effect, except for a single contrast that was significant. This, however, is only weak evidence in the context of the proposed effect. Our effect size estimates are consistently smaller than in the original study and closer to O’Donnell & Nelson [33] replication attempt (figure 4). We cannot rule out that we successfully replicated a very small effect for which we had not enough power to find statistical significance.

Some researchers have argued that there is always an effect of some, maybe even trivial, size (crud factor, e.g. [46]). In this case, one might wonder if the effect is relevant enough in real-world situations to warrant further investigation.

No matter how carefully designed and conducted a replication study is, it is but one further piece of evidence. No single replication can completely rule out the existence of the effect, nor is it able to provide final or absolute evidence for its existence. We think it is important—even more so in the light of the recent debate on the ‘replication crisis’—to conduct replications to add further data to the research literature upon which further studies and meta-analyses can be performed.

As the literature body grows, new statistical methods emerge and ever more data are collected, the synthesis of evidence through meta-analyses is vital to the sustainability of research. By providing our stimulus material and data openly to the research community, researchers are invited to re-analyse our data or re-run the study to collect more data before analysing the complete dataset.

O’Donnell & Nelson [33] have also shared their results from a replication attempt on study 3 openly and we were able to include their results when comparing the effect sizes from our replication attempt to the effect sizes of the original experiments.

Unfortunately, the original authors did not decide to openly share their material and data. We contacted both authors three times, but we were initially not able to obtain material or data from the original authors. It was only after we finalized the study and provided the first preprint, that we received the questionnaires used by the original authors. And it was only then that we discovered a major difference between the operationalization in the original study when compared to our replication attempt: the original study used not only words to prime participants in the ‘feeling’ condition but used also pictures related to these words. Since this was not described in the original paper, we did not include pictures in our priming condition. It seems reasonable to us that pictures might facilitate the priming and thus increases the effect of the priming task when compared to word-only priming. Future replication attempts should consider this information when trying to investigate the rounded price effect using a priming manipulation.

Our replication attempt is further limited regarding (i) the sample variation and (ii) the variation in stimuli. Our sample seems to be more homogeneous than the original sample, which might have led to a reduction in variance. Since further information on the demographics of the original Amazon MTurk sample is not available, we cannot conclude if the effect in question is dependent on specific sample characteristics. While household income or any personal experience with the product category could be seen as possible moderators from a theoretical point of view, any such influence would question the generalizability of the original findings. We have also included a second, different but similar product to our replication attempt since the originally used product might not be suitable for an investigation of the effect in our sample: as our pre-study revealed, digital camera binoculars are not a very attractive product—at least to German students aged between 22 and 25 years. A reviewer has raised the concern that products need to elicit positive feelings in order for the rounded price effect to take effect. This might be investigated in future studies by means of a systematic manipulation of the amount of positive feelings that are elicited by the products.

It has to be noted, however, the effect was originally reported as to be dependent only on the roundedness of the price and the cognitive versus emotional context of the purchase decision. If further context dependencies exist such as product attractiveness or personal characteristics to modulate the effect, the general nature of the rounded price effect and its simple moderation through a fit between context and roundedness are in question.

When researchers are interested in further investigation of the rounded price effect, the available replications highlight the need for large samples to detect the effect and the selection of products. As can also be seen in figure 4 the replication studies, i.e. ours and the replication attempt for study 3 by O’Donnell & Nelson [33], show smaller effect size estimates with narrower confidence intervals. This leads us to expect that the true effect size will be smaller and closer to the estimates from the replication attempts. We recommend at least 800 participants in future studies and a product that is more attractive to participants. It might also be interesting to see if the effect is more robust when using instructions rather than priming to operationalize the context similar to first experiments in the original article. To bolster the confidence in follow-up research, we also recommend pre-registration of the hypotheses of any further study on the effect [14,47]. This is of special importance when the effect of further moderators or covariates like the socio-economic status, age, culture and positive feelings are investigated.

Based on our findings and on the findings of the previous replication attempt, we suspect that the rounded price effect and the mediation through ‘feeling right’ as reported by Wadhwa & Zhang [1] is considerably smaller than originally reported. As always, no single or two replication studies suffice to finally rule out any small effect. Further experiments may explore the effect for products more appealing to participants and generate more estimates for the effect size as a basis for subsequent meta-analyses. The practical relevance of the possible effect for marketers would be worth the effort.

## Supplementary Material

p-Curve Disclosure Table

## Footnotes

1 Professor Wadhwa provided a copy of their questionnaire including the stimulus material only after the first preprint of this paper became available. Differences between the material used here and the original stimuli are discussed below.

2 The term might imply researchers are intentionally performing illegitimate procedures (similarly with the term ‘questionable research practices’), but it has to be stated that several of these techniques and analysis decisions, which allow for *p*-values just below the significance threshold even when the null is true, are common. Only in recent years have they been investigated as actually being harmful to the properties of significance testing and science in general. Thus, we prefer the terms ‘garden of forking paths’ (as used by [18]) or ‘researchers degrees of freedom’, which do not imply intentional behaviour.

3 The completed spreadsheet with data from the original study is available in our OSF repository.

4 In a previous version of this paper, that was first available as preprint, the selection of tests to include in both the *p*-curve and the R-Index analysis was not consistent. The paper, preprint and disclosure table in the appendix have been corrected accordingly to reflect the changes and now offer a corrected interpretation.

5 We received the inquired information only after the first preprint of this paper was uploaded. The discussion section was updated to consider the information received.

6 Pre-registration is also available at https://aspredicted.org/59vcd.pdf.

7 This affected primarily the cognition-priming if participants did not answer the calculus questions.

8 All stimulus material is available in the OSF repository.

## Data accessibility

All study data and stimulus material are available at an Open Science Framework (OSF) repository at https://osf.io/r942e/ (http://dx.doi.org/10.17605/OSF.IO/R942E) [48]. A preprint of this paper was uploaded to PsyArXiv (https://dx.doi.org/10.17605/OSF.IO/YD4RE).
