# Supplementary material for: Does it actually feel right? A replication attempt of the rounded price effect
Source: R Soc Open Sci. 2018 Apr 25;5(4):171127. doi: 10.1098/rsos.171127 (PMC5936890; doi:10.1098/rsos.171127)
Supplement: p-Curve Disclosure Table [file rsos171127supp1.pdf]

Study	Quoted text from original paper indicating prediction of interest to researchers	Study design	Key statistical result	Quoted text from original paper with statistical results	Results
1	We predicted that participants should show a stronger intention to purchase the bottle of champagne when it is priced at a rounded price as compared to when it is prices at a nonrounded price. In contrast, participants should show a stronger intention to purchase the calculator when it is priced at a nonrounded price as compared to when it is priced at a rounded price.	3 (price: rounded vs. high nonrounded vs. low nonrounded) × 2 (product type: hedonic vs utilitarian) *(reversing interaction)*	Four linear trends	Follow-up planned contrasts revealed that participants indicated higher purchase intention for the bottle of champagne (product type hedonic) in the rounded price condition ($M_{rounded}$ = 5.30) than in either of the two nonrounded price conditions ($M_{high\ nonrounded}$ = 4.07; t(173) = 2.03, p = .043; $M_{low\ nonrounded}$ = 3.77; t(173) = 2.55, p = .012). […] Conversely, follow-up tests revealed that participants reported lower purchase intention for the calculator (product type utilitarian) in the rounded price condition ($M_{rounded}$ = 4.43) than in either of the two nonrounded price conditions ($M_{high\ nonrounded}$ = 6.03; t(173) = 2.59, p = .01; $M_{low\ nonrounded}$ = 5.73; t(173) = 2.11, p = .036).	t(173) = 2.03<br>t(173) = 2.55<br>t(173) = 2.59<br>t(173) = 2.11
2	We predicted that when buying a camera for a family vacation, both the anticipated satisfaction and the perceived product performance (as measured by the perceived quality of the pictures) should be higher when the camera is priced at a rounded number. On the other hand, when the camera is purportedly bought for a class project, both the anticipated satisfaction and the perceived quality of pictures should be higher when the camera is priced at a nonrounded number.	2 (price: rounded vs. nonrounded) × 2 (consumption goal: hedonic vs. utilitarian) *(reversing interaction)*	Two linear trends (per DV)	As shown in figure 2A, planned contrasts revealed that when buying a camera for a family vacation (consumption goal-hedonic), picture quality was perceived to be higher in the rounded price condition (M = 5.27) than in the nonrounded price condition (M = 4.78; t(217) = 1.68, p = .047, one-tailed test). In contrast, when buying a camera for a class project (consumption goal-utilitarian), picture quality was perceived to be higher in the nonrounded price condition (M = 5.75) than in the rounded price condition (M = 5.03; t(217) = 2.47, p = .007, one-tailed test). [...] As shown in figure 2B, planned contrasts revealed that when buying a camera for a family vacation (consumption goal-hedonic), participants indicated greater anticipated satisfaction with the camera when it was priced at a rounded price (M = 5.26) versus a nonrounded price (M = 4.14; t(217) = 2.84, p = .005). In contrast, when buying a camera for a class project (consumption goal-utilitarian), participants reported greater anticipated satisfaction when the camera was priced at a nonrounded price (M = 5.80) versus a rounded price (M = 5.06; t(217) = 1.89, p = .060).	t(217) = 1.68<br>t(217) = 2.47<br>t(217) = 2.84<br>t(217) = 1.89
3	Drawing upon these findings, we propose that when the processing resources are constrained, purchase decisions are likely to be driven by feelings and therefore rounded versus nonrounded prices should lead to more favorable product evaluations. On the other hand, when the processing resources are available, purchase decisions are likely to be driven by cognition and thus nonrounded versus rounded prices should lead to more favorable product evaluations.	2 (price: rounded vs. nonrounded) × 2 (processing resouces: constrained vs unconstrained) *(reversing interaction)*	Two linear trends	Specifically, […] in the processing resources-constrained condition […], participants reported higher purchase intention for the pair of digital camera binoculars when it was priced at a rounded price (vs. nonrounded price; M = 4.25 vs. M = 3.19; t(173) = 2.11, p = .036). In contrast, in the processing resources-unconstrained condition […], participants indicated higher purchase intention for the pair of digital camera binoculars when it was priced at a nonrounded price (vs. rounded price; M = 4.19 vs. M = 3.06; t(173) = 2.28, p = .024).	t(173) = 2.11<br>t(173) = 2.28
4	To elaborate, priming with rounded (nonrounded) numbers should positively affect evaluations for a subsequent unrelated product, when the product information generates	2 (priming: rounded numbers vs. nonrounded numbers)	Two linear trends (per hypothesis)	More importantly, planned contrasts revealed that when the product message generated positive feelings, participants primed with rounded numbers indicated greater purchase intention for the shampoo than those primed with nonrounded numbers ($M_{rounded\text{-}pos.\ feeling}$ = 4.24	t(402) = 2.19<br>t(402) = -2.42<br>t(402) = 2.17

	positive feelings (cognitive reactions).<br>[…]<br>We predict that priming participants with nonrounded versus rounded numbers in one task should positively affect evaluations of an unrelated product when the product is endorsed by a strong argument, but not when it is endorsed by a weak argument.	× 4 (product: message: possitve feelings vs. negative feelings vs. strong argument vs. weak argument)<br>*(reversing interaction)*		vs. $M_{\text{nonrounded-pos. feeling}}$ = 3.31, t(402) = 2.19, p = .029). However, when the product message generated negative feelings, those primed with rounded numbers indicated lower purchase intentions than those primed with nonrounded numbers ($M_{\text{rounded-neg. feeling}}$ = 2.94 vs. $M_{\text{rounded-neg. feeling}}$ = 3.95; t(402) = -2.42, p = .016). In the argument-based product message conditions, participants primed with nonrounded numbers indicated greater purchase intention for the shampoo than those primed with rounded numbers when the product was endorsed with a strong argument ($M_{\text{nonrounded-strong argument}}$ = 5.13 vs. $M_{\text{rounded-strong argument}}$ = 4.20; t(402) = 2.17, p = .031). While this effect was not reversed, it was eliminated when the product was endorsed with a weak argument ($M_{\text{nonrounded-weak argument}}$ = 3.92 vs. $M_{\text{rounded-weak argument}}$ = 3.98; t(402) = -.15, p > .88).	t(402) = -0.15
5	We predict that this subjective experience of "feeling right" will mediate the rounded price effect.<br>*[The predictions of the rounded price effect as above are included in the p-curve analysis as the mediation depends on the existence of a direct effect.]*	2 (price: rounded vs. nonrounded) × 2 (prime: feelings vs. cognition)	Two linear trends<br>z-Test for Mediation	Specifically, […] participants in the feeling-prime condition indicated higher purchase intention for the product when it was priced at a rounded price (M = 4.14) as compared to when it was priced at a nonrounded price (M = 3.06; t(314) = 3.04, p = .003). In contrast, our results show that in the cognition-prime condition, participants reported greater purchase intention for the product when it was priced at a nonrounded price (M = 4.13) than when it was priced at a rounded price (M = 3.38; t(314) = 2.10, p = .037). […]<br>A Sobel (1982) test confirmed that the reduction of the effect was significant (z = 3.07, p = .002).	t(314) = 3.04<br>t(314) = 2.1<br>z = 3.07
